# Browning of Adipocytes: A Potential Therapeutic Approach to Obesity

**DOI:** 10.3390/nu15092229

**Published:** 2023-05-08

**Authors:** Vittoria Schirinzi, Carolina Poli, Chiara Berteotti, Alessandro Leone

**Affiliations:** 1 Endocrinology and Care of Diabetes Unit—Azienda Ospedaliero-Universitaria S. Orsola Malpighi, Alma Mater Studiorum University of Bologna, 40126 Bologna, Italy; 2 IRCCS—Azienda Ospedaliero-Universitaria S. Orsola Malpighi, Alma Mater Studiorum University of Bologna, 40126 Bologna, Italy; 3 PRISM Lab, Department of Biomedical and Neuromotor Sciences, Alma Mater Studiorum University of Bologna, 40126 Bologna, Italy; 4 International Center for the Assessment of Nutritional Status and the Development of Dietary Intervention Strategies (ICANS-DIS), Department of Food, Environmental and Nutritional Sciences (DeFENS), University of Milan, 20133 Milan, Italy

**Keywords:** brown adipose tissue, obesity, thermogenic nutraceuticals, thermogenesis, browning

## Abstract

The increasing prevalence of overweight and obesity suggests that current strategies based on diet, exercise, and pharmacological knowledge are not sufficient to tackle this epidemic. Obesity results from a high caloric intake and energy storage, the latter by white adipose tissue (WAT), and when neither are counterbalanced by an equally high energy expenditure. As a matter of fact, current research is focused on developing new strategies to increase energy expenditure. Against this background, brown adipose tissue (BAT), whose importance has recently been re-evaluated via the use of modern positron emission techniques (PET), is receiving a great deal of attention from research institutions worldwide, as its main function is to dissipate energy in the form of heat via a process called thermogenesis. A substantial reduction in BAT occurs during normal growth in humans and hence it is not easily exploitable. In recent years, scientific research has made great strides and investigated strategies that focus on expanding BAT and activating the existing BAT. The present review summarizes current knowledge about the various molecules that can be used to promote white-to-brown adipose tissue conversion and energy expenditure in order to assess the potential role of thermogenic nutraceuticals. This includes tools that could represent, in the future, a valid weapon against the obesity epidemic.

## 1. Introduction

Obesity is becoming one of the leading causes of death in the world, representing an increasingly alarming health emergency [1]. Obesity is recognized as a chronic disease caused by genetic, environmental, psychological and social factors; it is also considered a condition that increases the probability of developing a wide range of non-transmissible comorbidities, such as cardiovascular and metabolic disorders including type 2 diabetes and numerous forms of cancer [2]. One of the greatest challenges of this millennium is to halt the rapid increase in obesity worldwide, which should be considered a form of malnutrition on par with undernutrition, if not more critical. In order to achieve this goal, dietary prescriptions and physical activity are not enough. It is necessary to reform food systems, from the agricultural sector to large-scale distribution, ensure the availability and accessibility of healthy foods, invest in the promotion of educational programs, provide consumers with tools for making informed choices and encourage the population to engage in more physical activity. These are only some of the projects that would ideally be implemented soon, but they require significant political and economic commitment [3]. The scientific community is focusing on searching for alternative therapeutic strategies that accompany the refinement of modern technologies. The identification of BAT with its thermogenic potential may represent a valid way to tackle obesity, both by increasing energy expenditure and by modulating numerous metabolic targets [4,5,6]. The aim of this review is to address this hot topic by exploring the most recent studies in the literature and to examine a wide range of new bioactive dietary compounds that can expand and activate BAT, and to simultaneously promote the browning process in humans.

## 2. Adipose Tissue: Typologies, Role, Physiology

According to their antagonistic functions, two types of adipose tissue can be distinguished: the white adipose tissue (WAT), which stores excess energy, and the brown adipose tissue (BAT), which is specialized in dissipating energy via the production of heat. WAT represents the main form of storage of excess energy derived from food. The excess energy is stored as triglycerides and when necessary, is released in the form of three fatty acids and a glycerol. This feature guarantees the survival of an organism even during long fasting periods. In addition to its primary function as an energy reserve, WAT also has an important mechanical and insulating function, and protects the organs against trauma and cold. No less important is the endocrine function of WAT, which is now defined as an endocrine organ, being a source of the production of hormones and biologically active substances, including adipokines. It serves as a central node of inter-organ metabolic communication and a regulator of reproduction and satiety [1].

Similar to WAT, BAT synthesizes and secretes “batokines” such as fibroblast growth factors (FGFs), including FGF21, neuregulin 4, vascular endothelial growth factor (VEGF), and cytokines, such as interleukin 6 (IL-6). Given the relatively small amount of BAT present in humans, the endocrine potential of batokines is relatively unknown, but it is clear that factors secreted from BAT exert paracrine and autocrine functions. While the relative BAT mass in humans and rodents is small compared to other adipose depots, its relative contribution to metabolic health may be higher [7].

## 3. Distribution of Adipose Tissue

WAT can be broadly classified by location, largely defined as either subcutaneous (located under the skin) or visceral/omental (located intra-abdominally, adjacent to internal organs). In most lean, healthy individuals, WAT is confined to defined depots, but in certain conditions, such as obesity and lipodystrophy, WAT mass can increase ectopically in areas that may influence an individual’s susceptibility to comorbidities such as diabetes and atherosclerosis [8]. Such ectopic WAT areas are mostly located within the visceral cavity, and include intrahepatic, epicardial (between the heart and the pericardium), perivascular (surrounding major blood vessels), mesenteric fat (contiguous with digestive organs in the viscera), omental fat (an apron of fat that stretches over the intestines, liver, and stomach), and retroperitoneal fat (surrounding the kidneys). Visceral fat is highly metabolically active and is constantly releasing free fatty acids (FFA) into the portal circulation. As such, visceral fat content contributes to various features of the metabolic syndrome, such as hyperinsulinemia, systemic inflammation, dyslipidaemia, and atherosclerosis [9].

BAT is localized to distinct anatomical regions that have been well-characterized in rodents, helping them to survive cold temperatures [10]. While originally believed to be a depot exclusive to hibernating and small mammals, and present to some degree in human infants, adult humans have recently been shown to have functional and inducible levels of BAT. Thanks to an innovative technique called fluorodeoxyglucose positron emission tomography (FDG PET), combined with X-ray computed tomography, it has also been possible to identify metabolically active areas of BAT in adult humans that have total fat stores of between 1% and 2%, and that are localized primarily in the cervical, supraclavicular, interscapular, axillary, paravertebral, mediastinal, and upper abdominal regions [11]. Differently localized vessels in the organism present different proportions between WAT and BAT. This variability may be in conjunction with the different predominant functions of perivascular tissue according to localization. Large central vessels, for example, such as the aorta and its main ramifications, are mostly surrounded by brown adipose tissue, thus playing a key part in maintaining the central temperature within normal ranges. Peripherally increased perivascular adipose tissue, on the other hand, has been associated with increased insulin resistance [8]. Considering this, BAT has aroused great interest in scientific research, with the hope of exploiting its unique characteristics in the treatment of obesity-associated comorbidities.

## 4. Differences between White and Brown Adipocytes

The biology, the origins of BAT and the differences between WAT and BAT have been deeply reviewed in recent years. The most important differences are as follows: white adipocytes are spherical and unilocular cells (50–100 microns in diameter) with more than 90% of their volume represented by a single lipid droplet and a modest number of mitochondria. The spherical shape provides the adipocyte with a good way of accumulating volume in the smallest space and exporting energy molecules without the excessive anatomical breakdown of the tissue. White adipocytes derive from mesenchymal stem cells (MSCs), the precursors of adipocytes, but also cartilage, bone, and muscle cells. MSCs are directed towards the adipocyte line, in case of high energy intake. WAT has a great capacity for expansion, including hypertrophy and hyperplasia, in order to store large amounts of energy [12].

Brown adipocyte is defined as a multilocular cell, as it is composed of multiple, small lipid droplets and is rich in mitochondria with dense ridges, which give the tissue its brown colour, together with dense vascularisation. Mitochondria store energy as a proton gradient across the inner mitochondrial membrane, and this energy is used to synthesize adenosine triphosphate (ATP); when protons move along the gradient without producing ATP, the stored energy is dissipated as heat. This is due to the presence, exclusively in the brown adipocyte, of a specific protein: “the uncoupling protein 1 (UCP1)” in the inner mitochondrial membrane, which immunohistochemically is the defining protein marker of BAT. In contrast with WAT, the origin of brown adipocytes is related to the origin of skeletal muscle: both derive from specific cells of the dermomyotome, a portion of the mesoderm, that express myogenic factor 5 (Myf5) [13]. Another distinguishing feature of BAT is the innervation by the sympathetic nervous system, which is extremely reduced in WAT [14,15].

## 5. Conversion of White Adipocytes into Brown-like Adipocytes

**Figure 1:**
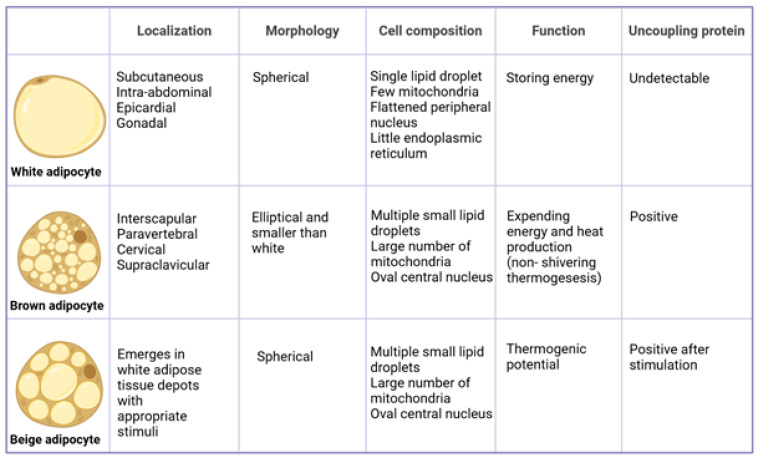
Overview of the main characteristics of white, brown, and beige adipocytes.

Adipose tissue is an extremely dynamic organ, capable of transforming in response to environmental or dietary stimuli. One of the possible ways to increase the presence of functional UCP1-rich cells in the adipose tissue is the conversion of white (pre)adipocytes into brown-like fat cells. This phenomenon is known as the “browning” of adipocytes or as the reversible and transdifferentiation of the adipose organ [16]. The browning process of WAT has become a key area of interest in research due to its role in fat burning; it is thus a potential useful strategy for treating obesity. The result is the appearance of dispersed masses of brown-like adipocytes in WAT, termed as beige or brite adipocytes. These cells possess similar characteristics to brown adipocytes, such as the multilocularised accumulation of lipids, a high mitochondrial content and an elevated UCP1 expression, along with factors that stimulate the transcriptional activity of thermogenic proteins. Nevertheless, brite adipocytes also display a distinct gene expression pattern that differs from both white and brown adipocyte profiles [15,16]. The morphology, location, origin and function of white, brown and beige adipocytes are reported in Figure 1.

At present, the mechanisms known to induce the browning of WAT in humans are cold exposure and β-adrenergic receptor stimulation. However, both these approaches are difficult to implement in human beings due to the non-specific nature of β-adrenergic agonists and the inability of humans to tolerate cold conditions for prolonged periods. Researchers in recent years have asked if it is possible to induce beige adipocyte differentiation without cold exposure and adrenergic stimulation. Recent studies would seem to offer answers: certain key genes, such as Proline Rich Domain Containing Protein 16 (PRDM16) [17] and Early B-cell Factor 2 (EBF2) [18], which appear to play a dominant role in programming brown adipocytes, have been identified [19]. The activation of these genes initiates a signal transduction cascade that culminates in the overexpression of UCP1 and other thermogenic proteins. Numerous studies have confirmed that, endogenously, the mechanisms responsible for classic BAT recruitment and WAT browning are identical, whereas exogenous molecules may selectively activate BAT thermogenesis or recruit brite adipocytes [20].

## 6. Thermogenesis and Thermogenin

In physiology, thermogenesis refers to a specific metabolic process that results in the production of heat by the body, particularly in adipose and muscle tissue. Heat is produced by the transformation of chemical energy via oxidative or catabolic processes. Thermogenesis is responsible for maintaining a constant internal temperature to cope with changing external climatic conditions and to ensure thermoneutrality: the ideal internal temperature for the body’s proper functioning [17]. Thermogenesis depends on both endogenous factors, genetic in nature, and exogenous factors, and therefore varies from individual to individual. An important exogenous factor affecting thermogenesis is caused by a cold environmental temperature, a metabolic process that leads to the production of heat by the body depending on how cold the environment is. Cold-induced thermogenesis (TIF) is divided into two types: ‘non-shivering thermogenesis’ and ‘shivering thermogenesis’. Non-shivering thermogenesis represents an increase in heat production by the body when exposed to cold, but is not associated with muscular contraction. It is caused by the increased activity of the sympathetic nervous system, which innervates BAT to a greater extent, and skeletal muscle. An alteration in BAT leads, in fact, to a high intolerance to cold in mammals. By contrast, ‘shivering thermogenesis’ requires the rhythmic isometric contraction of skeletal muscle, and it plays a less important role than ‘non-shivering’ thermogenesis; it takes over after it only [18,19]. BAT has an extraordinary ability to stimulate thermogenesis more than any other district of the body, thus increasing the total energy expenditure [20]. BAT heat production takes place in the mitochondria of brown adipocytes via the process of mitochondrial uncoupling, which involves the transmembrane protein UCP1 or thermogenin. It is responsible for ‘non-shivering thermogenesis’ and promotes the utilization of fatty acids that are not associated with ATP production but instead with heat production, reducing the efficiency of cellular respiration. The UCP1 is activated by fatty acids and is inhibited by nucleotides. Its thermogenic function is to dissipate the proton gradient generated by the respiratory chain and increase the permeability of the mitochondrial matrix, allowing proton dispersion [21]. This mechanism, used to produce heat, is a mammalian physiological response to low temperatures or excess nutrients from high-calorie diets. When sensory neurons are activated by cold, they transmit the stimulus to the brain, which in turn responds by increasing the activity of afferent nerves and releases noradrenaline. This neurotransmitter induces thermogenesis and lipid oxidation. UCP1-induced thermogenesis during exposure to cold is under the control of the hypothalamus, which integrates peripheral signals from skin thermoreceptors [22].

## 7. BAT-Secreted Factors with Potential Direct and/or Indirect Cardioprotective Effects

Current knowledge suggests the general beneficial effect of BAT activation regarding a reduction in the risk of cardiovascular disease (CVD) [23]. BAT secretes several molecules, which are collectively termed batokines. These batokines may alter the metabolism via autocrine, paracrine, and endocrine mechanisms, thus modifying BAT itself or acting remotely on other organs. Some BAT-secreted factors have potential direct and/or indirect cardioprotective effects via the modulation of the systemic metabolism. Summarizing, BAT generates heat via non-shivering thermogenesis, a process that helps to maintain body temperature and contributes to the overall energy expenditure. An increased energy expenditure may help to prevent excess weight gain and the development of obesity, which is a risk factor for CVD [24]. BAT consumes fatty acids as a primary fuel source for thermogenesis. By increasing the uptake and oxidation of fatty acids, BAT can help reduce circulating levels of triglycerides and lower the risk of atherosclerosis [25], a key factor in CVD. BAT also contributes to glucose homeostasis by taking up and metabolizing glucose. Improved glucose metabolism can help maintain insulin sensitivity and reduce the risk of developing type 2 diabetes, another CVD risk factor [26]. BAT secretes various hormones and cytokines, such as FGF21 and IL-6, which can have beneficial effects on metabolism, inflammation, and cardiovascular health. Some studies have suggested that BAT activation may help modulate blood pressure by promoting the release of nitric oxide, a vasodilator that helps relax blood vessels and lower blood pressure [27].

## 8. Browning Strategies: Cold, Physical Activity, and Adrenergic Agonists

Nowadays, it is known that UCP1 activation can be mediated by several factors: cold exposure, physical activity, fasting, nutraceutical foods, amino acids such as tyrosine (noradrenaline precursor), triiodothyronine (FT3), thyroxine (FT4), molecules that stimulate β-adrenergic receptors, and drugs that inhibit noradrenaline reuptake [6,28,29] (Table 1). The mechanism of action of the different browning strategies are summarized in Table 2.

Cold exposure (<20 °C) generates the production of noradrenergic signals that move from the periphery to the hypothalamus, via the orthosympathetic nerves that reach the BAT and release catecholamines such as noradrenaline [30]. Norepinephrine acts on the β-adrenergic receptors present on the cell membrane of brown adipocytes. These receptors activate adenylate cyclase, which catalyses the conversion of ATP to cyclic AMP (cAMP). cAMP in turn activates protein kinase A (PKA). Such a protein induces peroxisome proliferation, which activates the peroxisome proliferator-activated receptor gamma coactivator 1-alpha (PGC1α) receptor, which is the master regulator of the transcriptional cascade of UCP1 and other thermogenic genes [31]. To date, cold exposure has been proven to be the most effective strategy regarding BAT activation. Past studies on mouse models have shown that long-term cold exposure leads to an increase in adipocytes expressing UCP1, in both brown and beige adipose deposits, which can increase non-chill thermogenesis [19]. Studies on humans have shown that even a less prolonged exposure to the cold (17 °C for 2–6 h a day) is still capable of expanding BAT and increasing its functionality: the increase in BAT perfusion is directly associated with an increased total energy expenditure; and the increase in glucose uptake is due to the increased gene expression of the GLUT4 transporter in BAT. These effects of cold exposure on BAT are severely attenuated in individuals with excessive WAT deposition and the presence of insulin resistance [32,33]. Promising results are also emerging from the use of BAT activation strategies that exploit the modulation of adrenergic activity in the sympathetic nervous system. The stimulation of β-adrenergic receptors by adrenergic agonists [34], but also the combination of cold exposure with the infusion of sympathomimetics (isoprenaline and ephedrine) [35], can induce an increase in the levels of catecholamines released by the sympathetic nervous system and amplify thermogenesis. These results need further verification in order to clarify the modulation pathways [36]. Similar to cold exposure, exercise also promotes the upregulation of the activation markers of BAT (PGC1α and UCP1) and the stimulation of endocrine activators (cardiac natriuretic peptides, irisin and FGF21). Recent findings [37,38] show that exercise can have both an acute effect on BAT-releasing catecholamines via the activation of UCP1 in BAT and induce lipogenesis in WAT. The chronic effect of exercise on BAT concerns the discovery of specific myokines produced by skeletal muscle and released into the bloodstream during exercise. These myokines, identified in early 2014, are as follows: irisin, β-aminoisobutyric acid (BAIBA) and FGF21; these would appear to govern white adipocyte browning, independent of sympathetic nervous system stimulation [39,40].

**Table 2:** Mechanism of action of the different browning strategies: cold exposure, physical activity, adrenergic agonists and myokines (irisin, BAIBA and FGF21).

Intervention	Mechanism of Action	Bat Activation	Experimental Model	Ref.
COLD EXPOSURE		yes		
	UCP1 activation		human	[28,29,31]
	↑ production of noradrenergic signals		human	[30]
	↑ PGC1α		mouse human	[31]
	↑ UCP1 in BAT		human	[19]
	↑ BAT and ↑ its functionality		human	[32,33]
ADRENERGIC AGONISTS		yes		
	stimulate β-adrenergic receptors		human	[34]
	↑ levels of catecholamines and amplify thermogenesis		mouse	[35]
PHYSICAL ACTIVITY		yes		
	UCP1 activation		human	[37,38]
	↑ PGC1α		human	[37,38]
	releases catecholamines		human	[6,28]
	activates lipogenesis in WAT		human	[6,28]
MYOKINES				
Irisin	governs white adipocyte browning		human mouse	[29,30]
β-aminoisobutyric acid (BAIBA)	governs white adipocyte browning		human mouse	[29,30]
Fibroblast growth factor 21 (FGF21)	governs white adipocyte browning		human mouse	[29,30]

Abbreviations: UCP1: uncoupling protein 1, PGC1α: peroxisome proliferator-activated receptor gamma coactivator 1-alpha, BAT: brown adipose tissue, WAT: white adipose tissue, ↑: increase.

## 9. Thermogenic Nutraceuticals

**Figure 2:**
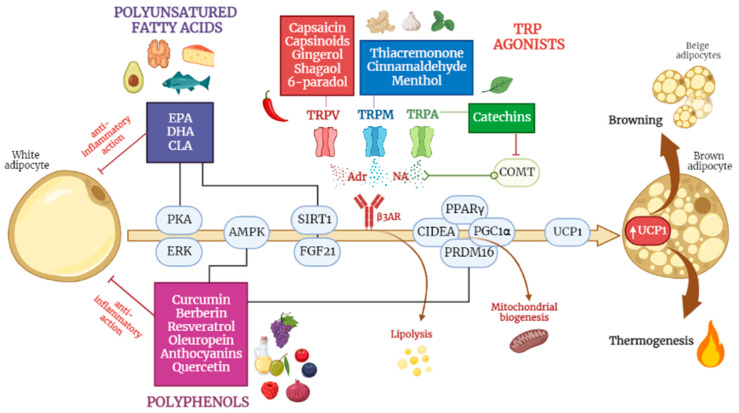
Molecular mechanisms involved in WAT browning and BAT activation mediated by thermogenic nutraceuticals. Each group of nutraceuticals, through different pathways, induces the expression of thermogenic genes (PPARγ, PRDM16, PGC1α, SIRT-1, CIDEA) leading to mitochondrial biogenesis and to the increase in UCP1 in white and brown adipocytes, which is responsible for the browning of WAT and enhancement of thermogenesis in BAT. TRP agonists, via the stimulation of adrenergic receptors, not only are crucial for the initiation of thermogenic pathways and beige differentiation, but also promote lipolysis. Polyunsaturated fatty acids and polyphenols, in addition, play an anti-inflammatory role in WAT.

Current evidence, both on animal and human models, indicates that several diet components may have beneficial effects on obesity by affecting BAT and energy metabolism, including polyunsaturated fatty acids, capsaicin and capsinoids, catechins, curcumin, resveratrol and berberine, oleuropein, anthocyanins, quercetin, gingerol, shogaol, 6-paradol, thiacremonone, cinnamaldehyde and menthol. The molecular mechanisms involved in WAT browning and the BAT activation mediated by thermogenic nutraceuticals are represented in Figure 2 and summarized in Table 3.

### 9.1. Polyunsatured Fatty Acids

Fish oil is rich in the polyunsaturated fatty acids (PUFA) eicosapentaenoic acid (EPA) and docosahexaenoic acid (DHA), which stimulate thermogenesis in BAT [41,42,43]. In the early 2000s, several studies in mouse models compared the effects of a diet enriched with *n*-3 PUFAs and a diet rich in *n*-6 PUFAs in order to study their impact on BAT. It turned out that fats from the *n*-3 series were much more effective in activating BAT than the *n*-6 series [44]. Therefore, subsequent research focused on the effects of *n*-3 PUFAs, especially EPA, and confirmed its anti-obesogenic effect, revealing that it can promote the browning of WAT. Rats fed with a supplementation of fish oil at a 20% EPA for 4 weeks showed an increased mRNA level of UCP1 in WAT [45]. Another study showed that supplementation with 27.5% fish oil, administered for one month, stimulates SNS-mediated mitochondrial and thermogenic activity in rats [46]. A diet enriched with a mixture of EPA and DHA increases the expression of UCP1 in BAT and reduces adipose accumulation via the induction of marked, non-shivering thermogenesis [47,48]. Further implications emerged from a study conducted in 2016, which revealed that a high-fat diet (HFD), enriched with fish oil (12% of total lipids) and administered to mice for 8 weeks, modulates BAT activation via epigenetic regulation [49]. EPA promotes both the adipogenesis of mature brown adipocytes [50] and the differentiation of pre-adipocytes into beige adipocytes, within WAT stores, particularly in the inguinal WAT [48]. It is important to note that the conversion of mature white adipocytes to beige adipocytes was achieved via supplementation with EPA in human adipocyte cultures [51] and that the same effect was not observed in rodents [43].

### 9.2. Capsaicin and Capsinoids

Capsaicin is an alkaloid found in chilli peppers that is mainly concentrated in the seeds of the fruit and is the main component responsible for the spicy flavour. Capsaicin has a role in preventing the obesogenic effects of diet, due to its ability to increase energy expenditure by activating BAT [52]. Capsaicin implements BAT function via several signalling pathways, primarily via the activation of Transient Receptor Potential Vanilloid 1 channels (TRPV1), which stimulate the central nervous system to produce catecholamines involved in thermogenesis. Capsaicin seems to regulate the epigenetic expression of the transcription factors involved in WAT browning [53,54]. Important evidence has also emerged from studies on humans: Yoneshiro et al. [55] examined the acute effect of an oral ingestion of a single dose of capsinoids (9 mg) on energy expenditure, in relation to BAT activity in humans; this was measured by 18F-FDG PET, a classical imaging technique used to visualize BAT activation. The ingestion of capsinoids resulted in a 3-fold increase in energy expenditure in the BAT-positive group compared to the BAT-negative group. Furthermore, the chronic intake of capsinoids (9 mg/day in capsule form) for 6 weeks promoted BAT activity and reduced fat mass after cold exposure, even in human subjects with low BAT activity. In 2018, Camps et al. [56] tested the same treatment used in mice in 2016 [57] on humans. They gave a group of normal-weight individuals a dose of capsinoids (12 mg), combined with exposure to cold (14.5 °C). The subjects underwent, before and after exposure, both 18F-FDG PET and indirect calorimetry, in order to assess the total energy expenditure. The results showed that capsinoids increased energy expenditure in BAT-positive participants and, when combined with cold, increased fat oxidation, improved insulin sensitivity and increased HDL-cholesterol [58,59].

### 9.3. Green Tea Catechins

Green tea consumption is associated with weight loss and the modulation of fat metabolism and energy expenditure [60,61]. Catechin supplementation for 8 weeks reduced the mass of perirenal WAT and increased the expression of mRNA coding for UCP1 in BAT, compared to the control group in rats [62]. Yan and al. [63] showed that the oral administration of catechins (100 mg/kg body weight) for 4 weeks significantly reduced the total fat mass (subcutaneous and visceral) and liver size in rats fed with HFD. No browning of the WAT was observed, but fatty acid oxidation in the BAT increased twofold [63]. Several experiments testing the effects of catechin supplementation on rodents were also conducted on humans. The habitual intake of green tea (>300 mg catechins/day) was found to be as effective as in rats in terms of reducing body weight and preventing weight regain [64]. Similar results were obtained in a study on subjects with metabolic syndrome, where catechin supplementation additionally improved the lipid profile and hypertension [65]. The consumption of 300 mg/day of Epigallocatechin Gallate (EGCG) or 200 mg of caffeine [66] for 3 days in subjects with obesity increased postprandial fat oxidation, but not the total energy expenditure [67]. A 12-week treatment with green tea extracts containing 583 mg catechins resulted in a small but significant reduction in body fat (3–5%) in healthy, normal-weight subjects [68]. A more recent study [69] suggested that catechins also act as inhibitors of catechol-O-methyltransferases (COMT), enzymes that degrade catecholamines. The inhibition of COMTs results in the increased metabolism of catecholamines, including noradrenaline, which, as we have seen, is involved in thermogenesis and fat oxidation processes.

### 9.4. Curcumin

Curcumin is the most abundant and active element within the turmeric rhizome; it is a widely studied polyphenol with antioxidant and anti-inflammatory properties, also known for its anti-obesity effects [70]. Isolated WAT cells of obese rats, in response to treatment with 20 μM of curcumin for 6–8 days, showed an increase in the thermogenic markers of BAT and in the hormone-sensitive lipase (HSL), which is responsible for triglyceride mobilization and lipolysis in WAT. These results suggest curcumin’s role in improving the lipid profile in an obese condition [71]. The administration of high doses of curcumin (45 mg/kg body weight) causes an increase in energy expenditure via the induction of mitochondrial biogenesis in mice [72]. Experimental evidence on humans is still limited. However, one significant study in the literature stands out; this was conducted in 2019 on 60 participants [73,74], with the aim of assessing the effects of curcumin supplementation on cardiovascular risk factors among overweight adolescents. It was a randomized controlled trial involving 60 girls aged 13–18 years, randomly assigned to the intervention or control group. The intervention group was supplemented with 500 mg of curcumin (in bioavailable form) daily for 10 weeks, while the control group received a placebo. At the same time, they were asked to undergo a mild weight loss or weight maintenance diet, depending on the degree of overweight or obesity. The results revealed that curcumin supplementation induced significant improvements compared to the control group, such as a reduction in the body mass index, a reduction in waist circumference and hip circumference, and reduction in the triglyceride/HDL ratio; meanwhile, it induced an increase in the HDL cholesterol. However, clinical studies on a larger population and with a longer duration are needed to confirm the results of these studies [73,74] and to test the WAT darkening effects observed in rodents also in humans.

### 9.5. Resveratrol

Resveratrol is a polyphenol naturally contained in grape skins and other vegetables such as red fruits, cocoa and peanuts [75]. The supplementation of 30 mg/kg body weight of resveratrol in mouse models for 8 weeks showed a significant reduction in fat mass, plasma glucose concentrations and total cholesterol, compared to the control group [76]. There was also evidence of increased UCP1 expression in both BAT and skeletal muscle [77]. This is attributed, at least in part, to the ability of resveratrol to activate upstream AMPK, which promotes the production of PGC1α and Sirtuin 1 (SIRT1); these, in turn, promote mitochondrial biogenesis and WAT browning [78]. The effect of resveratrol on the neo-formation of beige adipocytes is thought to be mediated by the phosphorylation of AMPK, as the deletion of this protein in mice abolishes the browning effects of resveratrol [79]. A recent study [80] revealed the additional signalling pathways exerted by resveratrol. The results reported regarding the cell cultures and animal models suggest that high doses of resveratrol (100 and 200 μM) induce epigenetic regulatory mechanisms. Polyphenol can up-regulate the expression of the genes that code for the proteins involved in WAT adipogenesis, such as fatty acid synthase (FAS), the sterol response element-binding protein (SREBP1), lipoprotein lipase (LPL) and hormone-sensitive lipase (HSL) [81]. Overall, resveratrol oversees antiadipogenic and anti-inflammatory effects, which are dependent and independent of BAT activation; however, evidence supporting its thermogenic effects in humans is currently lacking. This is due to the poor bioavailability of polyphenolic compounds [82], so it is difficult to achieve effective doses that are able to stimulate BAT through food alone. Diet alone is not sufficient in order to mimic the efficacy of resveratrol observed in rodents.

### 9.6. Berberine

Berberine is an alkaloid compound found in the roots, rhizomes and bark of certain plants of the genus Berberis, primarily known for its anti-cancer activity [83]. Zhang et al. [84] proved that the intraperitoneal administration of berberine, administered at 5 mg/kg/day for 4 weeks in obesity-induced mice, played a key role in increasing energy expenditure and the mobilization of lipids. More specifically, the thermogenic effects attributed to berberine concerned the increase in the mitochondrial content and thermogenic markers in BAT (UCP1, PGC1α, Cidea). The increase in BAT was detected via PET-CT, while the increase in the resting energy expenditure was quantified by measuring the oxygen consumption (VO_2_) and carbon dioxide release (VCO_2_) before and after treatment. The total expenditure increased by 20%.

An interesting finding, which did not emerge from studies on the other phytochemical compounds, is that this treatment caused a significant reduction in the respiratory quotient, suggesting that berberine causes a shift in cell metabolism, taking energy from the oxidation of fatty acids, rather than from carbohydrates. Furthermore, mice berberine not only stimulates BAT activity but also induces the browning of the inguinal WAT, probably via the phosphorylation of AMPK, which leads to the increased expression of UCP1 and PGC1α [85]. An in vitro study was recently conducted on human preadipocytes, and it was found that the thermogenic effect induced by berberine on WAT depends on the recruitment of the AMPK-PRDM16 axis: the activation of AMPK could lead to DNA demethylation and thus upregulate the expression of PRDM16, which acts as a master regulator of the transdifferentiation of white adipocytes into beige adipocytes; this occurs via the direct modulation of the transcription factors PPARγ and PGC1α [86,87].

### 9.7. Other Nutraceutical Compounds

Many other dietary polyphenolic compounds have been found, in rodent studies, to influence BAT thermogenesis and WAT browning: oleuropein, anthocyanins, quercetin, and structural analogues of capsaicin (i.e., menthol, cinnamaldehyde, allyl and benzyl isothiocyanates, which are the spicy elements found in mustard and wasabi, and thiacremonone). Some of these compounds have also shown promising results in humans.

#### 9.7.1. Oleuropein

Oleuropein is the main polyphenol contained in olive leaves and fruits, and is responsible for the pungent and bitter taste of raw olives. Olive leaf extract (3 mg of oleuropein, injected intravenously in mice for 7 weeks) increases UCP1 content in BAT by activating SIRT1, PPARγ and PGC1α. In addition, it stimulates the secretion of adrenalin and noradrenalin via the activation of TRP channels [88]. Oleuropein aglycone (the absorbed form of oleuropein) appears to be able to attenuate diet-induced obesity by supporting the expression of thermogenic genes and genes related to mitochondrial biogenesis in the BAT of overfed mice [89], and to promote the browning of adipose tissue via mesenchymal stem cells in humans [90].

#### 9.7.2. Anthocyanins

Anthocyanins are the polyphenols mainly contained in red fruits, grapes, black soy, and red beans. Anthocyanins are known first and foremost for their strong antioxidant power, but they have also been re-evaluated for their thermogenic action in stimulating adrenalin secretion and energy metabolism in humans; meanwhile, in rodents, they exert an anti-obesity action that is related to BAT activation [91,92]. In humans, the intake of 150 mg/day of an extract of Aronia melanocarpa, a particular type of blueberry rich in anthocyanins, procyanidins and other flavonoids, increases the surface body temperature and plasma adrenalin levels, suggesting that it has a stimulating effect on the SNS [93]. Numerous studies have also shown that long-term treatment with cyanidin-3-glucoside, which is found in raspberry and mulberry extract, increases UCP1 expression and mitochondrial biogenesis during the adipogenic differentiation of brown and white preadipocytes [94,95]. Similarly, black soybean peel extract upregulates the expression of thermogenic genes in BAT, induces WAT browning and increases the lipid respiration quotient, thus preventing visceral fat accumulation in mice on a hyperlipidic diet [96].

#### 9.7.3. Quercetin

Quercetin is a flavonoid contained in a wide variety of fruits (apples, grapes, olives, citrus fruits, berries) and vegetables (tomatoes, onions, broccoli, and capers). It has a thermogenic effect in mice and an ability to modulate the gut microbiota in mice. Obese mice fed a HFD supplemented with 1% quercetin for 16 weeks lost weight and had reduced plasma cholesterol levels, compared to mice fed a HFD alone [97]. The improvement in obesity is explained by the ability of quercetin to increase the expression of thermogenic genes in BAT (UCP1, PGC1α. FGF21), as well as and genes coding for β-adrenergic receptors and AMPK; this results in increased non-excitation thermogenesis. The increase in AMPK also suggests that quercetin may predispose these signalling pathways to WAT browning [98]. Other studies point out the flavonoid’s ability to modulate the intestinal microbial composition of mice. Quercetin reduces the ratio of Firmicutes to Bacteroidetes in the microbiota of HFD-fed mice, improving the obesity-related dysbiosis picture. In fact, a eubiotic microbiota allows greater energy extraction from the diet via the increased production of short-chain fatty acids, which have been found in the faeces of mice supplemented with quercetin [97].

#### 9.7.4. Analogues of Capsaicin

Structural analogues of capsaicin include menthol, which is a cyclic monoterpene alcohol obtained from peppermint, the activator of TRPM8 receptors [99,100], cinnamaldehyde, a pungent compound that increases UCP1 expression in human white adipocytes [101] and is found in cinnamon, allyl and benzyl isothiocyanates, which are spicy elements found in mustard, ginger and wasabi (Japanese horseradish), and finally, thiacremonone, a sulphur compound isolated in garlic [102]. The compounds just mentioned can activate TRPV1 and TRPA1 channels (also belonging to the TRP receptor family, but activated by cold, mechanical stimuli and cooling nutritional compounds), which modulate thermoregulation. The combination of sub-effective doses of capsaicin, cinnamaldehyde and menthol induce the “brite” phenotype in the differentiation of the 3T3-L1 cells and subcutaneous white adipose tissue of HFD-fed obese mice [103]. The intervention prevented adipose tissue hypertrophy and weight gain, and enhanced the thermogenic potential, mitochondrial biogenesis, and overall activation of brown adipose tissue. These changes, observed in vitro as well as in vivo, were linked to the increased phosphorylation of kinases, AMPK and ERK. In the liver, this combination treatment enhanced insulin sensitivity, improved the gluconeogenic potential and lipolysis, prevented fatty acid accumulation and enhanced glucose utilization. Finally, it is worth mentioning that gingerol, shogaol and 6-paradol, which are all contained in ginger root, activate TRPV1 channels [104,105,106]. An interesting study in humans looked at a type of ginger native to West Africa (black ginger), from which the extract Kaempferia parviflora is derived. The administration of 100 mg/day of this extract appears to increase energy expenditure in BAT-positive subjects, and the activation of brown adipocytes is detectable via FDG-PET after exposure to cold [107].

**Table 3:** Mechanism of action of the thermogenic nutraceuticals examined.

Thermogenic Nutraceuticals	Dose	Mechanism of Action	Experimental Model	Ref.
EPA DHA		simulates thermogenesis in BAT	mouse human	[41,42,43,44]
		↑ expression of UCP1 in BAT,	mouse	[47,48]
		↓ adipose accumulation via the induction of marked, non-shivering thermogenesis,	mouse	[36,37]
		promotes the adipogenesis of mature brown adipocytes	mouse	[50]
		promotes the differentiation of pre-adipocytes into beige adipocytes, particularly in the inguinal WAT	mouse	[48]
Capsaicin		activates TRPV1 channels: implements BAT function	in vitro and pre-clinical studies	[53,54]
		regulates the epigenetic expression of the transcription factors involved in WAT browning	in vitro and pre-clinical studies	[53,54]
Capsinoids	9 mg/day in capsule form for 6 weeks	promotes BAT activity and reduces fat mass	human	[55]
	12 mg combined with exposure to cold (14.5 °C)	↑ energy expenditure and, when combined with cold, ↑ fat oxidation, ↑ insulin sensitivity and ↑ HDL-cholesterol	human	[56,57,58]
Catechins	for 8 weeks	↓ mass of perirenal WAT, ↑ expression of mRNA coding for UCP1 in BAT	rat	[62]
	100 mg/kg body weight for 4 weeks	↓ total fat mass (subcutaneous and visceral) and liver size, fatty acid oxidation in the BAT increased twofold	rat	[63]
	>300 mg catechins/day	↓ body weight and prevents weight regain	human	[64]
		inhibits catechol-O-methyltransferases	human	[69]
Curcumin	20 μM for 6–8 days	↑ in thermogenic markers of BAT and in hormone-sensitive lipase (HSL),	isolated WAT cells of obese rats	[71]
	45 mg/kg of body weight	↑ energy expenditure via the induction of mitochondrial biogenesis	mouse	[73]
	500 mg (in bioavailable form) daily for 10 weeks	↓ in body mass index, waist circumference and hip circumference, and triglyceride/HDL ratio, and ↑ HDL cholesterol	human	[73,74]
Resveratrol	30 mg/kg of body weight for 8 weeks	↓ fat mass, plasma glucose concentrations and total cholesterol	mouse	[76]
		↑ UCP1 expression	mouse	[77]
		activates upstream AMPK, which promotes the production of PGC1α, and SIRT1, which promotes mitochondrial biogenesis and WAT browning	mouse	[78]
		up-regulates the expression of genes coding for proteins involved in WAT adipogenesis (FAS, SREBP1, LPL and HSL)	human	[81]
Berberine	5 mg/kg/day for 4 weeks	↑ energy expenditure and the mobilization of lipids	mouse	[84]
		stimulates BAT activity, and induces browning of the inguinal WAT	mouse	[85]
Oleuropein	3 mg injected intravenously for 7 weeks	↑ UCP1 content in BAT by activating SIRT1, PPARγ and PGC1α, stimulates the secretion of adrenalin and noradrenalin via the activation of TRP channels	mouse	[88]
Oleuropein aglycone (the absorbed form of oleuropein)		attenuates diet-induced obesity by supporting the expression of thermogenic genes and genes related to mitochondrial biogenesis in the BAT of overfed mice	mouse	[89]
		promotes the browning of adipose tissue from mesenchymal stem cells in humans	in vitro	[90]
Anthocyanins	150 mg/day of an extract of Aronia melanocarpa	↑ surface body temperature and plasma adrenalin levels, suggesting that it has stimulating effect on the SNS	human	[91]
	long-term treatment with cyanidin-3-glucoside, such as raspberry and mulberry extract	↑ UCP1 expression and mitochondrial biogenesis during adipogenic differentiation of brown and white preadipocytes	rat	[92,93]
	black soybean peel extract	↑ the expression of thermogenic genes in BAT, induces WAT browning and increases the lipid respiration quotient, preventing visceral fat accumulation on a hyperlipidic diet	mouse	[94]
Quercetin	HFD supplemented with 1% quercetin for 16 weeks	reduces plasma cholesterol levels	mouse	[95]
		↑ the expression of thermogenic genes in BAT (UCP1, PGC1α, FGF21) and genes coding for β-adrenergic receptors and AMPK	in vitro	[96]
Menthol		activates TRPM8 receptors	in vitro	[98]
Cinnamaldehyde		↑ UCP1 expression	in vitro	[97]
Gingerol, Shogaol, 6-Paradol		activates TRPV1 channels	in vitro mouse	[102,103,104]
	100 mg/day of Kaempferia parviflora	↑ energy expenditure in BAT-positive subjects; activates brown adipocytes	human	[107]

BAT: brown adipose tissue; UCP1: uncoupling protein 1; WAT: white adipose tissue; TRPV1: transient receptor potential vanilloid 1; HSL: hormone-sensitive lipase; AMPK: adenosine monophosphate-activated protein kinase; PGC1α: peroxisome proliferator-activated receptor gamma coactivator 1-alpha; SIRT1: Sirtuin 1; FAS: fatty acid synthase; SREBP1: sterol response element-binding protein; LPL: lipoprotein lipase; PPAR: peroxisome proliferator-activated receptor; TRP: transient receptor potential; SNS: sympathetic nervous system; FGF21: fibroblast growth factor 21; TRPM8: transient receptor potential cation channel subfamily M member 8; ↑: increase, ↓ decrease.

## 10. Conclusions

To face the increasing rate of obesity and the associated metabolic risks, the scientific community is focusing on the search for alternative therapeutic strategies and pharmacological treatments, which currently are rather limited and correlated with numerous side effects. New therapeutic strategies accompany the refinement of modern technologies, such as PET-CT, which has made it possible to re-evaluate the role of adipose tissue. Thanks to these recent imaging techniques, the BAT, with its thermogenic potential, may represent a valid strategy for combating obesity both by increasing energy expenditure and by modulating numerous metabolic targets [108,109,110]. Today, the goal is to find strategies that can expand and activate BAT, and simultaneously promote the browning process of adipose tissue in order to increase energy expenditure. One such strategy is cold exposure, which has long been known as the most effective way of activating BAT and browning WAT. An alternative method is the use of adrenergic agonists that directly stimulate the sympathetic nervous system to activate BAT. Recent findings regarding the role of metabolites produced during exercise, such as irisin, β-aminoisobutyric acid, FGF21 and natriuretic peptides, which could act as BAT activators, are also very promising. In recent years, research has focused on identifying food and nutraceutical components that have similar effects to cold exposure. The mechanisms of action of several nutraceutical factors have been characterized, and innovative methods for BAT activation and WAT darkening, without causing the side effects associated with previous strategies, have been discovered. The most consistent findings concern the role of capsinoids and catechins in green tea. The consistent supplementation of capsinoids in high doses (>10 mg) is effective in promoting thermogenic activation in humans, which has important implications regarding the prevention and therapeutic treatment of obesity. The habitual consumption of green tea (~100 mg/kg body weight of catechins) appears to promote BAT recruitment and induce weight loss, at least in animals. Further studies in humans are needed in order to understand whether the beneficial effects of green tea are due to catechins or to other components. A widely studied dietary compound is fish oil, particularly EPA. Although there is some controversy as to the effective dose, there is evidence that fish oil consumption activates multiple signalling pathways in order to increase BAT and trigger WAT darkening. Several polyphenolic compounds, such as resveratrol, curcumin, oleuropein, quercetin and berberine, have been recognised as thermogenic activators and play an important role in the transdifferentiation of white adipocytes into beige adipocytes. Thermogenic activation, in animals, was evidenced with the supplementation of high doses of polyphenols (>0.1% or 100 mg/kg body weight), which are considered to have super-physiological effects in humans. A major challenge for future experiments will be to overcome the poor bioavailability of polyphenol molecules in food.

To conclude, lifestyle modification interventions remain the first line of intervention in the treatment of obesity. However, considering the difficulties generally experienced by obese patients in terms of following a low-calorie diet and exercising on a regular basis, this therapy can be enhanced via the combination of innovative strategies. In view of these considerations, a personalized dietary program could be complemented by the proper supplementation of thermogenic nutraceuticals, which are molecules capable of increasing energy expenditure by enhancing BAT’s own thermogenesis. Further studies in humans are needed to confirm the effects of the proposed nutritional factors, which could represent, in the future, a promising intervention for the treatment of obesity and associated comorbidities.

## Figures and Tables

**Table 1:** Factors responsible for activation of UCP1.

UCP1 activation	Cold Exposure	[6,28,29]
	Physical Activity	
	Fasting	
	Nutraceutical Foods	
	Amino Acids such as Tyrosine (Noradrenaline precursor)	
	Triiodothyronine (Ft3), Thyroxine (Ft4)	
	Molecules that stimulate β-adrenergic receptors and drugs that inhibit noradrenaline reuptake	

## Data Availability

Not applicable.

## Abbreviations

ADRB3	β-3 adrenoceptor
AMP	Adenosine monophosphate
AMPK	adenosine monophosphate-activated protein kinase
ATP	adenosine triphosphate
BAIBA	β-aminoisobutyric acid
BAT	brown adipose tissue
cAMP	cyclic adenosine monophosphate
COMT	catechol-O-methyltransferase
CREB	cAMP response element binding protein
CVD	cardiovascular disease
DHA	docosahexaenoic acid
DIT	diet-induced thermogenesis
EGCG	epigallocatechin gallate
EPA	eicosapentaenoic acid
ERK	extracellular signal-regulated kinase
FAS	fatty acid synthase
FGF21	fibroblast growth factor 21
FT3	free triiodothyronine
FT4	free thyroxine
GLUT	glucose transporter
HFD	high fat diet
HSL	lipase sensitive hormone
IL-6	interleukin 6
LPL	lipoprotein lipase
MSCs	mesenchymal stem cells
Myf5	myogenic factor 5
NPY	neuropeptide Y
NST	non-shivering thermogenesis
OA	oleuropein
PET	positron emission tomography
PGC1α	peroxisome proliferator–activated receptor gamma coactivator 1-alpha
PKA	protein kinase A
PPAR	peroxisome proliferator-activated receptor
PRDM16	proline rich domain-containing protein 16
SIRT1	Sirtuin 1
SREBP1	sterol response element-binding protein
SNS	sympathetic nervous system
TIF	cold-induced thermogenesis
TLR	toll-like receptor
TNFα	tumour necrosis factor alpha
TRPA1	transient receptor potential cation channel subfamily A member 1
TRPM8	transient receptor potential cation channel subfamily M member 8
TRPV1	transient receptor potential vanilloid 1
UCP1	uncoupling protein 1
VEGF	vascular endothelial growth factor
WAT	white adipose tissue
